# Oral Metastasis From Colorectal Adenocarcinoma: Report of a New Case and a Scoping Review

**DOI:** 10.1155/crid/9978193

**Published:** 2025-02-08

**Authors:** Gioele Gioco, Romeo Patini, Denise Marchesini, Cosimo Rupe, Edoardo Staderini, Alberta Lucchese, Eugenio De Corso, Carlo Lajolo

**Affiliations:** ^1^Head and Neck Department, Catholic University of the Sacred Heart, Rome, Italy; ^2^Multidisciplinary Department of Medical-Surgical and Dental Specialties, University of Campania “Luigi Vanvitelli”, Naples, Italy

**Keywords:** case report, colorectal adenocarcinoma, oral metastasis, scoping review

## Abstract

**Objectives:** This study is aimed at describing an unusual case of oral metastasis from colorectal adenocarcinoma and at performing a scoping review analyzing the available literature on such cases.

**Materials and Methods:** We present a rare case of oral metastasis from colorectal adenocarcinoma in a 37-year-old Italian woman. Clinical oral examination showed the presence of a swollen lesion in the vestibular gingiva of the right mandibular region, associated with Grade III mobility of the second premolar. Radiographic examination revealed a radiolucency apical to the first molar and second premolar associated with the rhizolysis of these teeth. Furthermore, we conducted a scoping literature review of the PubMed, Web of Science, and Scopus databases in line with the Preferred Reporting Items for Systematic Reviews and Meta-Analyses extension for scoping reviews (PRISMA-ScR) guidelines.

**Results:** The second premolar was extracted, and an incisional biopsy of the osteolytic lesion was performed for histological and immunohistochemical examination, which revealed an adenocarcinoma fragment with mucinous characteristics and a morphology and immunophenotype compatible with caudal-related homeobox transcription factor 2 (CDX2) and cytokeratin (CK) 20. In conclusion, the lesion was indicative of oral metastasis from colorectal adenocarcinoma. Furthermore, the review of the existing literature in the English language yielded 112 cases of oral metastases from colorectal adenocarcinoma, which were included in our analysis.

**Conclusions:** Although oral metastasis from colorectal adenocarcinoma has been reported previously, it is a rare manifestation.

**Clinical Relevance:** Because of the rarity of oral metastasis and possible variations in the clinical and histological presentations, correct diagnosis can be challenging and requires careful clinical and histopathological evaluations with adjuvant immunohistochemical studies.

## 1. Introduction

Metastasis is a process involving the dissemination of cancer cells from a primary tumor to other organs and is one of the fundamental causes of cancer-related mortality [1].

Although metastasis to the oral cavity is usually rare [2], its detection is crucial because dissemination to the oral cavity could be the first sign of the disease in some cases. However, in most cases, patients presenting with oral metastases have already received a primary tumor diagnosis, and the presence of oral metastasis is an indication of the terminal stage of the disease [3].

According to the available literature, oral metastasis is mostly observed in patients aged 50–70 years, with no differences between the sex [4]. The posterior region of the jaw is the most commonly affected site in the oral cavity.

The diagnosis represents a critical issue due to aspecificic symptoms and signs: swelling, pain, bleeding, paresthesia, tooth mobility, halitosis, regional lymphadenopathy, mandibular nerve involvement and numb-chin syndrome, cortical expansion of the jawbones, ulceration, trismus, exophytic growth, and rarely pathological fracture [5–7].

Moreover, early diagnosis of jaw metastases is even more challenging because the lesions may not have a radiographic presentation during the initial stages of the disease [8].

The primary tumors that most commonly metastasize to the oral cavity include breast cancer in females and lung cancer in males. Nevertheless, other types of tumors may occasionally cause oral metastasis (e.g., kidney and bone cancers) [4]. Metastases from other primary tumors are rare, and their incidence has been reported in a few studies, usually case reports. Particularly, oral metastasis from colorectal carcinoma is very rare.

This study is aimed at describing a rare case of oral metastasis from colorectal adenocarcinoma and at performing a scoping review of similar such cases in the literature for clinical application.

## 2. Methods

### 2.1. Case Presentation

This case report was written following the CARE checklist (Supporting Information 1), and the patient signed an informed consent form to allow the publication of this paper. A 37-year-old woman was referred to our Oral Medicine Department for evaluation of hypoesthesia of the lower lip and gingival swelling associated with mobile posterior teeth of the right side. The medical history of the patient included Crohn disease treated with infliximab and left colon adenocarcinoma (T3 N0 M0), which was diagnosed in 2017 and treated surgically by hemicolectomy. In 2020, the patient was affected by local adenocarcinoma recurrence treated by proctectomy, radiotherapy, and chemotherapy with capecitabine.

In 2021, a PET-CT revealed multiple bone, lung, and brain metastasis. Thus, the patient underwent repeated cycles of radiotherapy and chemotherapy with FOLFOX–panitumumab and denosumab.

**Figure 1:**
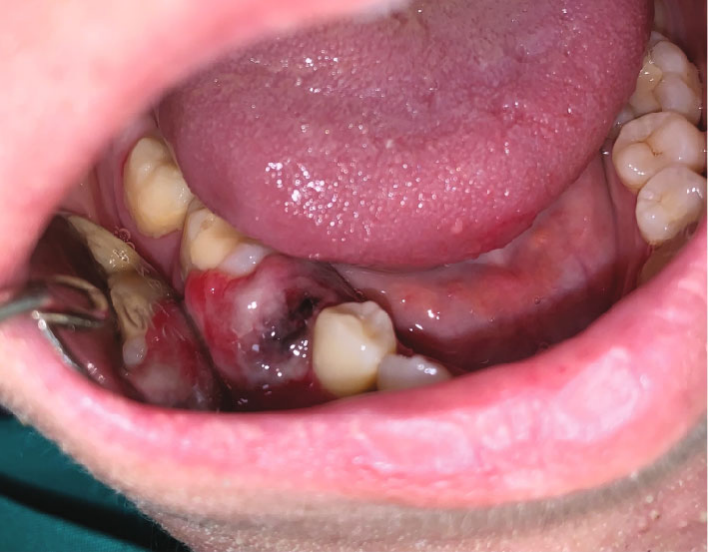
Clinical features of oral metastasis from colon adenocarcinoma.

The clinical oral examination confirmed the presence of a swollen lesion in the vestibular gingiva of the right mandibular region (Quadrant IV), buccal to the second premolar and first molar, which was associated with Grade III mobility of the second premolar. The lesion measured approximately 2.5 cm × 2.5 cm × 2 cm (Figure 1).

**Figure 2:**
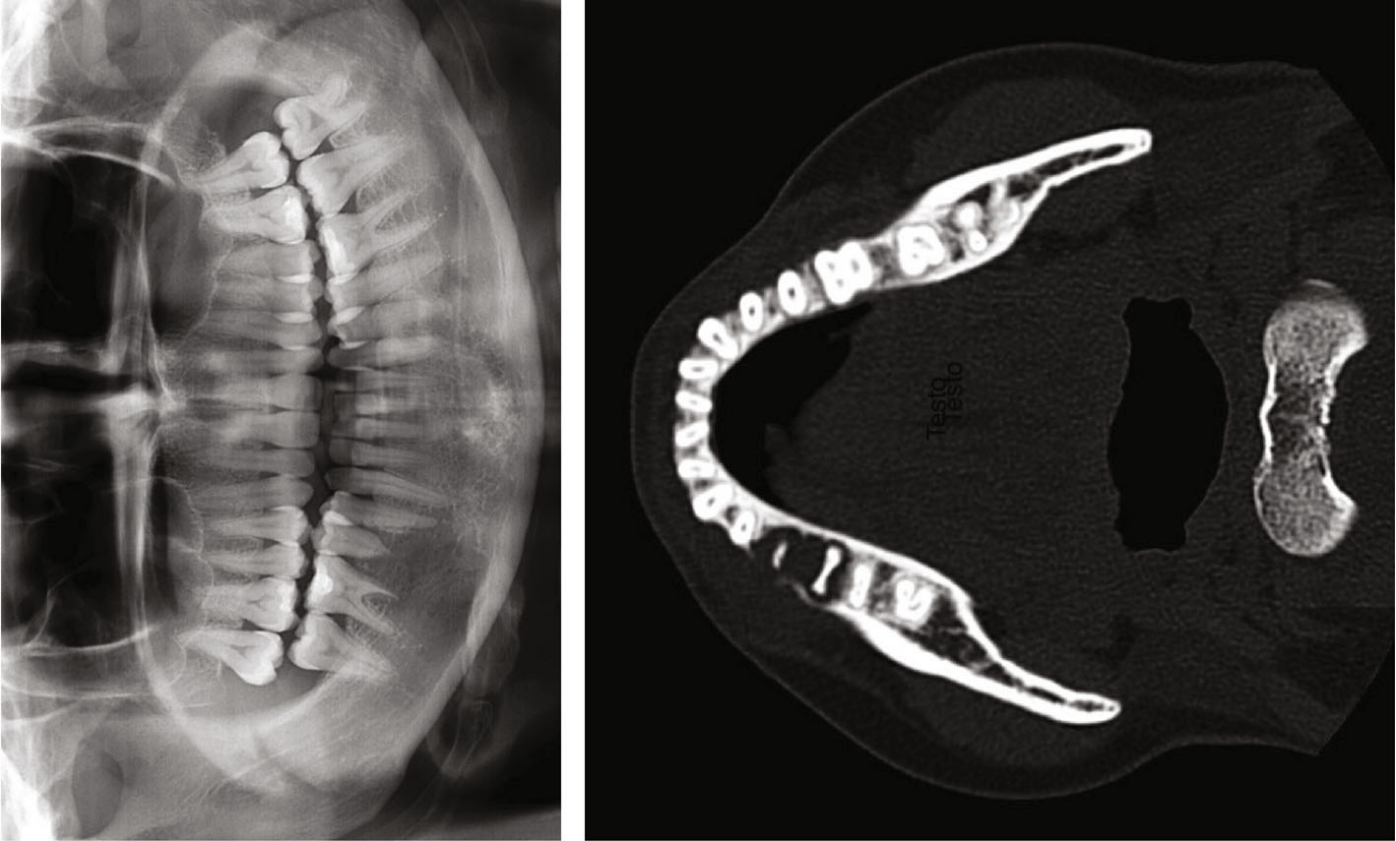
Radiological examination of oral metastasis from colon adenocarcinoma showing osteolytic lesion surrounding the first molar and second premolar associated with rhizolysis of these teeth.

Radiographic examination revealed a radiolucency apical to the first molar and second premolar associated with previous rhizolysis of these teeth (Figure 2). Although the second premolar was not affected by caries, it was extracted under local anesthesia due to the enhanced tooth mobility, and an incisional biopsy of the osteolytic lesion under the extraction site alveoli was performed for histological examination. Moreover, immunohistochemical analysis was performed to identify adenocarcinoma metastases.

Histopathological examination revealed an adenocarcinoma fragment with mucinous characteristics. Immunohistochemistry analysis showed a morphology and immunophenotype compatible with caudal-related homeobox transcription factor 2 (CDX2) and cytokeratin (CK) 20 and negative results for CK7, *α*-smooth muscle actin, p63, and S100. In conclusion, the lesion was indicative of oral metastasis from colorectal adenocarcinoma.

Due to the advanced stage of the oncological diseases, the molecular features, and the age of the patient, it was decided to continue with the systemic cytotoxic first-line treatment according to the FOLFOX–panitumumab regimen.

Nevertheless, 6 months after, a further PET-CT revealed an increased uptake in the right hemimandible besides the other already known metastasis, showing only a partial response to the chemotherapy. Thus, the patient started a novel chemotherapy with FOLFIRI–bevacizumab treatment.

During the monthly follow-up visits, the clinical examination revealed an edematous, nonpainful swelling of increasing size. Moreover, 8 months after the oral metastasis diagnosis, the first molar was extracted due to expansive growth of the metastasis and increased mobility of the tooth (Grade III), and the patient started palliative radiotherapy for mandibular and vertebral metastases. However, the treatment was interrupted due to high toxicity, and the de Gramont chemotherapy was started. Nevertheless, 10 months after the diagnosis of oral metastasis, the patient died due to the oncologic disease.

### 2.2. Scoping Review

A scoping review of the published cases of oral metastasis due to colorectal adenocarcinoma was performed to analyze the available literature on such cases, including our case report. This scoping review was conducted in accordance with the Preferred Reporting Items for Systematic Reviews and Meta-Analyses extension for scoping reviews (PRISMA-ScR) guidelines [9, 10] (Supporting Information 2).

The inclusion criteria were full papers, English language, observational clinical studies (case reports and prospective and retrospective (cohort and case–control) studies), and randomized clinical trials, if available. Patients affected by oral metastasis due to colorectal adenocarcinoma were included in this review, particularly those with a histopathological diagnosis.

The exclusion criteria were the lack of a histological diagnosis of oral metastasis in patients with colorectal carcinoma and non-English studies.

### 2.3. Search Methods for Identification of Studies, Selection of Studies, and Data Extraction

A comprehensive and systematic electronic search of Medline (via PubMed), Scopus, and the Cochrane Central Register of Controlled Trials was conducted from database inception. The final search was conducted on February 10, 2023.

Electronic searches were performed using a combination of the following Medical Subject Headings terms and free-text words: ((Colon Cancer) OR (Colon Adenocarcinoma)) AND (Oral metastases).

A manual search was conducted for articles published in the following journals: *Oral Oncology*; *Clinical Oral Investigations*; *Journal of Oral Pathology and Medicine*; *Oral Surgery, Oral Medicine, Oral Pathology, and Oral Radiology*; *Head and Face Medicine*; and *Oral Diseases*.

Additionally, the bibliographies of all papers were checked to select other potentially relevant studies.

The studies' eligibility was independently assessed in a nonblinded and standardized manner by two reviewers (G.G. and D.M.). In the first round, records were screened by the titles and abstracts. In the second round, the full texts of the eligible papers were read. Only the articles that fulfilled the inclusion criteria were included in this scoping review. If there was disagreement regarding the selection of a paper, it was evaluated by a third reviewer (C.L.) for the final decision.

General data regarding the included studies and specific information regarding oral metastasis due to colorectal adenocarcinoma were collected using a customized data extraction form, including the year of publication, age, sex and nationality of patients, localization of the primary tumor, size and clinical aspect of the lesions, intraoral metastasis site, symptoms, radiological examination, histopathological features, immunohistochemical patterns, time elapsed from initial symptoms to first evaluation, time to achieve a final diagnosis, time from primary tumor detection to oral metastasis, follow-up period, and time from oral diagnosis to death. Missing data were not considered and thus not included in the analysis.

## 3. Results

### 3.1. Results of the Search and Study Selection

**Figure 3:**
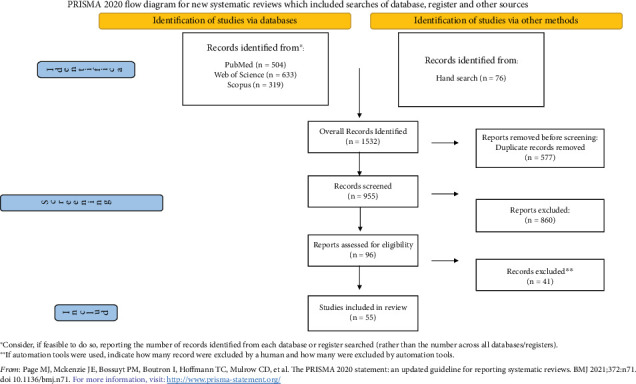
Flow diagram according to PRISMA-ScR.

The review process identified 1532 articles: PubMed search, 504 records; Scopus search, 319 records; and Web of Science search, 633 records. After excluding studies that did not fulfil the inclusion criteria and removing duplicates, we identified 96 publications for full-text reading. All the full texts were obtained, and a critical evaluation was performed. A manual search of the reference lists accompanying the published articles yielded three additional eligible articles; thus, 55 of the 96 papers were retrieved for this review. Details regarding the full flow of information presented in the PRISMA-ScR format are reported in Figure 3.

A summary of the full-length articles excluded and the reasons for their exclusion are reported in Supporting Information (Supporting Information 3).

### 3.2. Study Characteristics and Summary of Results

Among the 55 included studies and our case report, this review retrieved data of 112 patients with oral metastasis from colorectal adenocarcinoma. The characteristics of the included studies and the demographic and clinical data of the included patients are reported in Supporting Information 4. Out of 112 patients, 54.3% were male and 45.7% were female, with an average age of 66.7 years. According to the available clinical reports, the lesions were located mainly in the mandible (32 in the mandible vs. 12 in the maxilla); the soft tissue was affected in 32 cases, both soft tissue and bone in 21, and only bone in 14. Particularly, oral metastases from colorectal adenocarcinoma affected the gingiva (*n* = 37; 42.5%), alveolar mucosa (*n* = 8; 9.2%), tongue (*n* = 3; 3.5%), cheeks (*n* = 3; 3.5%), and bone (*n* = 36; 41.4%). The other 25 cases were not described and were then excluded from the statistical analysis.

Clinically, the mean size of the oral metastasis lesions was 34.6 mm (range: 10–100 mm) and mainly occurred as solitary, painless, exophytic, nontender, soft, reddish, irregular-shaped lesions. No differences were detected in the clinical oral metastases between the colon and rectal adenocarcinoma cases.

Although oral metastasis is the last manifestation of the oncological disease in most cases, the oral lesions were diagnosed before the primary tumor in 12 cases (10.7%). Data regarding the treatment were available only for 44 patients out of 112: chemotherapy alone and chemotherapy in combination with radiotherapy were administered in 12 and 8 cases, respectively. Radiotherapy alone was administered in 10 cases, while surgery was performed in five cases. The mean follow-up period was 9.2 months (range: 2–60 months), and the mean survival time was 8.7 months (range: 1–49 months).

All 112 patients underwent histopathological analysis, which revealed adenocarcinoma pathology.

Immunohistochemical analysis was performed in 29 cases, all of which were positive for CK20. Carcinoembryonic antigen (CEA) was reported in nine studies and all of which were positive. On the other hand, some markers were not univocally determined; 19 of 20 cases were positive for CDX2, while 14 of 19 were negative for CK7.

## 4. Discussion

Colorectal adenocarcinoma is the third most frequent cancer, especially in developed countries [11, 12], with an incidence of 1,931,590 new cases worldwide. Moreover, it is one of the leading causes of cancer-related death, with the World Health Organization reporting 935,173 deaths.

Colorectal adenocarcinoma is one of the most frequent cancers that cause metastases. Approximately 20% of the patients present metastasis in the advanced stage of the cancer [13], whereas approximately 60% develop distant metastases within 5 years [14].

Numerous mechanisms have been implicated in the metastasis process; nevertheless, the most likely mechanism is the hematogenous spread to the liver, followed by the lungs and bone, as well as the peritoneum, which represents the most commonly involved site of metastasis.

The oral cavity represents a rare site for metastasis; lung cancer and breast cancer are the two neoplasms that usually cause oral metastasis [15–17]. Oral metastases originating from colorectal cancers are rare and have a poor prognosis. In this review, we identified 112 cases of oral metastases due to colorectal adenocarcinoma, with major involvement of the mandible. Usually, oral involvement is rare and occurs more frequently in the mandibular bone, with only a few gingival cases published. The exact mechanism of oral metastasis remains unknown, and the most validated theory seems to be related to the vertebral system of the veins, which can be a route for hematogenous dissemination, thus skipping the neck region and allowing oral metastasis to occur, according to the Batson theory [7, 18].

Diagnosis is difficult because of the rare presentation and atypical clinical and radiographic manifestations. The oral metastatic lesions can present as radiopaque or sclerotic areas or appear similar to an infected cyst or osteomyelitis [19]. In most cases, the lesions present as a lytic radiolucent lesion with ill-defined margins [20].

Furthermore, symptoms are very nonspecific, such as swelling, pain, bleeding, paresthesia, tooth mobility, loosening of teeth, halitosis, regional lymphadenopathy, mandibular nerve involvement and numb-chin syndrome, cortical expansion of the jawbones, ulceration, trismus, exophytic growth, and rarely pathological fracture [6]. Owing to its nonspecific clinical presentation, the differential diagnosis might be challenging because it can be misdiagnosed clinically as odontogenic infection, trauma, benign odontogenic tumors and cysts, reactive lesions, primary tumors (e.g., salivary gland neoplasms), or systemic diseases (e.g., amyloidosis, sarcoidosis, multiple sclerosis, or neurological manifestation of a nonmetastatic malignancy) [16]. Furthermore, radiological examinations of metastatic tumors within the bone show irregular, ill-defined, and destructive radiolucent lesions that could be mistaken for infected odontogenic cysts, advanced periodontal bone loss, osteomyelitis, or a primary bone or odontogenic malignancy. Tumor cells rarely stimulate osteoblastic bone deposition, resulting in a mixed radiopaque/radiolucent appearance radiologically.

For a correct diagnosis, a biopsy of the suspected lesion is mandatory along with ancillary techniques such as immunohistochemical examination, which is a useful tool for identifying the primary site. The main histological differential diagnosis of metastatic adenocarcinoma is a primary intraoral tumor, especially those originating from the salivary glands. The differentiating factor for colorectal adenocarcinoma is a typical CK20+/CK7− profile in the immunohistochemical analysis. The CK20 labeling confirms the origin of the cells as digestive, urinary, or from a Merkel tumor, while CK7 is expressed in the simple epithelium of the breasts, lungs, mesothelium, female genital tract, and urinary bladder, with reduced expression in the gastric and intestinal mucosa [21]. CK20+/CK7− profile is typical for colorectal adenocarcinoma and has been used to differentiate it from adenocarcinomas of other origins (e.g., female genital tract, lungs, breasts, and liver) [22].

Another important marker is the CEA, a glycoprotein produced by a high percentage of colorectal cancers, which helps determine the malignant characteristics of a tumor. It can be quantitatively detected in serum, and its level is useful as a disease marker. Elevated levels are a poor prognostic sign and correlate with reduced overall survival after surgical resection of colorectal carcinoma [23]. Other immunohistochemical markers used for the diagnosis of oral metastasis from colorectal adenocarcinoma are shown in Table 1.

Metastatic lesions in the oral cavity grow rapidly and are responsible for pain, masticatory disturbances, and bleeding, and the treatment choice is a debatable topic. In most cases, the tumor is treated with surgical resection either alone or combined with radiation therapy and chemotherapy. Specifically, surgical resection is recommended when only oral metastasis is present; however, if the tumor is widely disseminated, palliative radiotherapy is recommended. Moreover, since oral metastasis is the last manifestation of the disease in most of the cases, treatment options are sometimes restricted to palliation to preserve the quality of life [45].

## 5. Conclusion

Although metastases from colon adenocarcinoma to oral cavity are uncommon (112 reported cases), they should be considered in the differential diagnosis of many osteolytic lesions. The malignant features should be assumed when unusual rapid growing lesions appear in the oral cavity. Malignance should be highly suspected when unusually rapid growth is evident in the oral lesion. To achieve a correct diagnosis, a clinical examination should be conducted, and past medical history should be evaluated. Nevertheless, pathology and immunohistochemical examination are necessary. Due to poor prognosis, the therapeutic options should include palliative therapy to preserve quality of life.

## Figures and Tables

**Table 1:** Immunohistochemical features retrieved of oral metastasis from colorectal adenocarcinoma.

**Marker**	**Staining**	**Description**
CK20 [2, 3, 16, 24–44]	++	Keratin 20 is a Type I cytokeratin in mature enterocytes and is specifically found in the gastric and intestinal mucosa.
CK7 [3, 16, 24–28, 30, 32–34, 36, 38, 40, 41, 44]	−	Keratin 7 is a Type II keratin. It is specifically expressed in the epithelia lining the cavities of the internal organs and in the gland ducts and blood vessels, specifically in ovarian, lung, breast, and bladder epithelia.
CK5/6 [16, 39]	++	Keratins 5 and 6 are a Type II keratin and are expressed primarily in basal keratinocytes in the epidermis.
CK19 [25]	++	Keratin 19 is a Type I keratin expressed in epithelial cells.
CDX2 [2, 3, 16, 27–42, 44]	++	The CDX2 protein is a homeobox transcription factor expressed in the nuclei of intestinal epithelial cells.
TTF1 [3, 25, 26, 30, 32, 33, 36]	−−	Thyroid transcription factor 1 (TTF-1) is a protein that regulates transcription of genes specific for the thyroid and lung.
CEA [24, 28, 38, 39, 44]	++	CEAs are glycosyl phosphatidyl inositol (GPI) cell surface–anchored glycoproteins and serve as functional colon carcinoma L-selectin and E-selectin ligands, which may be critical to the metastatic dissemination of colon carcinoma cells.
CA19-9 [27]	++	Carbohydrate antigen (CA 19-9) is a tumor-associated mucin glycoprotein antigen and is present in epithelial tissues of the pancreas, biliary ductal cells, stomach, gall bladder, colon, endometrium, salivary glands, prostate, normal pancreatic juice, and bile.
P16 [39]	++	p16 protein competes with cyclin D for binding to CDK4. This inhibits the ability of the cyclin D-CDK4 complex to phosphorylate Rb (retinoblastoma) protein, thus causing cell cycle arrest at late G1 phase.
P63 [39]	++	Tumor protein p63 is a member of the p53 family of transcription factors and is a key regulator of epidermal keratinocyte proliferation and differentiation.
Alpha-SMA [28]	−−	Cytoplasmic actins are part of the microfilament system of cytoskeletal proteins. Smooth muscle actin is found in vascular walls, intestinal muscularis mucosae, and muscularis propria and in the stroma of various tissue.
S-100 [28]	−−	S100 protein is a dimer and belongs to a calcium binding group of proteins. The protein is expressed in neural crest–derived tissues, chondrocytes, adipocytes, myoepithelial cells, dendritic cells of lymphoid tissue, Langerhans cells, and T lymphocytes.
CD117 [28]	−−	CD117 (KIT) is a Type III receptor tyrosine kinase operating in cell signal transduction in mast cells, some hematopoietic stem cells, germ cells, melanocytes, and Cajal cells of the gastrointestinal tract.
Synaptophysin [16]	−−	The protein is a synaptic vesicle glycoprotein and it is present in neuroendocrine cells.
Chromogranin A [16]	−−	Chromogranin A or parathyroid secretory protein 1 (gene name CHGA) is a member of the granin family of neuroendocrine secretory proteins, and it is located in secretory vesicles of neurons and endocrine cells (pancreatic beta cells, chromaffin cells of the adrenal medulla, paraganglia, enterochromaffin-like cells).
Villin [44]	++	Villin is a microfilament-associated, actin-binding cytoskeletal protein normally expressed in cells with highly specialized, brush border–type microvilli such as enterocytes and cells of the proximal renal tubules.
Inhibin [44]	−−	Inhibin is a gonadal hormone that downregulates FSH synthesis and inhibits FSH secretion. Inhibin is produced in Sertoli cells in the testis and in granulosa cells in the ovary.

*Note:* −−, negative in most cases (< 10% of cases exhibit positive staining); ++, positive in most cases (> 90% of cases exhibit positive staining); +, positive in most cases (50%–90% of cases exhibit positive staining); −, negative in most cases (10%–50% of cases exhibit positive staining).

## Data Availability

The data that support the findings of this study are available from the corresponding author, C.R., upon reasonable request.

## Supporting Information

Supporting Information 1 CARE checklist.

Supporting Information 2 PRISMA-ScR checklist.

Supporting Information 3 Articles excluded from this review and reasons for their exclusions.

Supporting Information 4 Characteristics of patients affected by oral metastasis from colorectal adenocarcinoma.
