# Supplementary material for: Oral Metastasis From Colorectal Adenocarcinoma: Report of a New Case and a Scoping Review
Source: Case Rep Dent. 2025 Feb 8;2025:9978193. doi: 10.1155/crid/9978193 (PMC11830109; doi:10.1155/crid/9978193)
Supplement: Supporting Information 3 — Articles excluded from this review and reasons for their exclusions. [file 9978193.f3.docx]

**Supplementary Files 3.** Articles excluded from this review and reasons for their exclusions.

| **Article** | **Reason for exclusion** |
| --- | --- |
| Ivy et al. 1936 | Article not available |
| Kronfeld et al. (1940) | Article not available |
| Piazzolla et al. (1940) | Article not available |
| Salmon et al.(1944) | Article not available |
| Cameron et al. (1947) | Article not available |
| Holland et al. (1953) | No colon cancer |
| Cash et al. (1961) | Article not available |
| Hatziotis et al. (1973) | No colon cancer |
| Zagarelli et al. (1973) | Post-mortem study |
| Perlmutter et al. (1974) | No colon cancer |
| Ooya et al.(1975) | Gardner's syndrome and mandibular osteoma |
| Solomon et al. (1975) | Article not available |
| Kim et al. (1979) | No colon cancer |
| Nishimura et al. (1982) | No oral metastasis |
| Schwartz et al. (1988) | No data regarding type of tumor |
| Piattelli et al. (1990) | Article not available |
| Sanchez Aniceto et al. (1990) | No colon cancer |
| Kim et al. (1990) | Article not available |
| Batsakis et al. (1991) | Article not available |
| Hirshberg et al (1993) | No colon cancer |
| Micali et al. (1994) | No data regarding histological examination |
| Bentley et al. (1997) | No oral metastasis |
| Pruckmayer et al. (1998) | No colon cancer |
| Tomikawa et al. (2001) | No colon cancer |
| Fukuda et al. (2002) | No colon cancer |
| Ahn et al. (2004) | Article not available |
| Jarrosson et al. (2005) | Article not in English |
| Nithyanand et al. (2006) | No data regarding primary tumor |
| Tamiolakis et al. (2007) | No cancer colon |
| Moure et al. (2008) | Article not in English |
| Kawamura et al. (2008) | No colon cancer |
| Barnes et al. (2009) | Review article |
| Gomes et al. (2009) | No data regarding primary tumor |
| Muttagi et al. (2011) | No colon cancer |
| Santini et al. (2012) | No oral metastasis |
| Wang et al. (2013) | No oral metastasis |
| Murillo et al. (2013) | No colon cancer |
| Qiu et al. (2013) | No colon cancer |
| Woo et al. (2015) | No colon cancer |
| Vatandoust et al. (2015) | Review article |
| Surya et al. (2015) | Review article |
