# Supplementary material for: Oral Metastasis From Colorectal Adenocarcinoma: Report of a New Case and a Scoping Review
Source: Case Rep Dent. 2025 Feb 8;2025:9978193. doi: 10.1155/crid/9978193 (PMC11830109; doi:10.1155/crid/9978193)
Supplement: Supporting Information 4 — Characteristics of patients affected by oral metastasis from colorectal adenocarcinoma. [file 9978193.f4.docx]

**Supplementary File 4.** Characteristics of patients affected by oral metastasis from colorectal adenocarcinoma.

| Case presentation | Author | Year | Country | Gender | Race | Age | Primary tumor | Site | Size (mm) | Treatment | Follow-up (mos) | Survival time (mos) | Time to onset (mos) |
| --- | --- | --- | --- | --- | --- | --- | --- | --- | --- | --- | --- | --- | --- |
| 1 | Castigliano ^24^ | 1954 | USA | F | W | 56 | rectum | right jaw, molar and premolar areas and the lower half of the ascending ramus of the mandible | 50 | radiotherapy and surgery | 6 | 6 | first metastases |
| 2 | Hàgglund ^25^ | 1959 | Sweden | F | \ | 77 | colon | temporomandibular joint | \ | surgery and radiotherapy | 12 | 32 | 24 |
| 3 | Clausen ^26^ | 1963 | Denmark | M | \ | 60 | sigmoid colon | gingiva around 3.2 | 25x20x16 | \ | 4 | 4 | 66 |
| 4 | Meyer ^27^ | 1965 |  | F | \ | 70 | rectum | mandible, body and ramus | \ | \ | \ | \ | \ |
| 5 | Meyer ^27^ | 1965 |  | F | \ | 64 | rectum | mandible, body, ramus, buccal mucosa, floor | \ | \ | \ | \ | \ |
| 6 | Meyer ^27^ | 1965 |  | F | \ | 78 | rectum | maxilla, palate, alveolar mucosa | \ | \ | \ | \ | \ |
| 7 | Levy ^28^ | 1974 | \ | F | W | 80 |  | left mandibular premolar area | \ | \ | 1 | 1 | 6 |
| 8 | Moffat ^29^ | 1976 | \ | \ | \ | 56 | rectum | lower gum margin posteriorly on the right | 12x10 | cyclical chemotherapy | \ | \ | 36 |
| 9 | Rentschler ^30^ | 1982 | Canada | M | W | 73 | rectum | lingual aspect of the left posterior lower gingiva | 40x30 | radiotherapy | 12 | \ | 27 |
| 10 | Rusthoven ^31^ | 1983 | Canada | F | W | 45 | rectum | right lower molar | \ | chemotherapy and radiation | 2 | 2 | 10 |
| 11 | Rusthoven ^31^ | 1983 | Canada | M | W | 65 | rectosigmoid colon | two right upper teeth | 30 | chemotherapy and radiotherapy | 8 | 5 | 36 |
| 12 | Tsianos ^32^ | 1985 | \ | M | \ | 80 | rectum | mandibular left premolar tooth area | \ | surgery | \ | \ | first metastases |
| 13 | Naylor ^33^ | 1989 | \ | F | W | 65 | sigmoid colon | adjacent to the mandibular right second bicuspid | 10x7x3 | palliative radiation | \ | \ | 60 |
| 14 | Nitzan ^34^ | 1989 | Israel | F | \ | 75 | rectosigmoid colon | left retromolar region | 20x20 | \ | \ | \ | first metastases |
| 15 | Davidson ^35^ | 1989 | UK | M | \ | 68 | upper rectum | posterior third of the tongue, right side | 25 | radiotherapy | 5 | 5 | 36 |
| 16 | Bhutani ^36^ | 1992 | \ | M | B | 57 | right colon | hard palate | 15 | chemotherapy | \ | \ | 24 |
| 17 | Govind Babu ^37^ | 1996 | India | M | \ | 75 | colon | left side of the jaw | \ | chemotherapy | \ | \ | together |
| 18 | Hsu ^38^ | 2001 | China | F | \ | 79 | rectum | right cheek | 50 | radiotherapy | \ | 1 | 49 |
| 19 | van der Waal ^18^ | 2003 | UK | F | \ | 70 | colon | hard palate | \ | \ | 6 | 6 | 43 |
| 20 | Mason ^39^ | 2005 | USA | M | \ | 73 | sigmoid colon | right parotid gland | 61x57 | no therapy | \ | \ | first metastases |
| 21 | Alvarez-Alvarez ^40^ | 2006 | \ | M | \ | 62 |  | right anterior mandibular gingiva | 40 | chemotherapy and palliative radiotherapy | 9 | 9 | 6 |
| 22 | Kuttan ^41^ | 2006 | USA | F | W | 62 | colon | alveolar mucosa of the jaw | 15x5 | palliative radiation | 4 | 4 | first metastases |
| 23 | Bodner ^42^ | 2006 | Israel | M | \ | 67 | rectum | jaw, between 11 and 21 | \ | chemotherapy | 14 | \ | \ |
| 24 | Lim ^43^ | 2006 | Korea | \ | \ | \ | colo-rectum | jaw | \ | \ | \ | \ | \ |
| 25 | Lim ^43^ | 2006 | Korea | \ | \ | \ | colo-rectum | jaw | \ | \ | \ | \ | \ |
| 26 | Lim ^43^ | 2006 | Korea | \ | \ | \ | colo-rectum | jaw | \ | \ | \ | \ | \ |
| 27 | Spinelli ^44^ | 2006 | Italy | M | \ | 72 | rectosigmoid colon | palate | 20 | \ | \ | \ | 60 |
| 28 | Chen ^7^ | 2008 | \ | M | \ | 64 | rectum | left mandibular ramus | \ | carbamazepine for neuralgia; palliative cheradiotherapy | 6 | 6 | \ |
| 29 | Kawamura ^45^ | 2008 | Japan | F | \ | 51 | rectum | right fifht to sixth interdental gingiva | 10x15 | \ | \ | 2 | 1 |
| 30 | Hirshberg ^16^ | 2008 | \ | M | \ | \ | \ | \ | \ | \ | \ | \ | \ |
| 31 | Hirshberg ^16^ | 2008 | \ | M | \ | \ | \ | \ | \ | \ | \ | \ | \ |
| 32 | Hirshberg ^16^ | 2008 | \ | M | \ | \ | \ | \ | \ | \ | \ | \ | \ |
| 33 | Hirshberg ^16^ | 2008 | \ | M | \ | \ | \ | \ | \ | \ | \ | \ | \ |
| 34 | Hirshberg ^16^ | 2008 | \ | M | \ | \ | \ | \ | \ | \ | \ | \ | \ |
| 35 | Hirshberg ^16^ | 2008 | \ | M | \ | \ | \ | \ | \ | \ | \ | \ | \ |
| 36 | Hirshberg ^16^ | 2008 | \ | M | \ | \ | \ | \ | \ | \ | \ | \ | \ |
| 37 | Hirshberg ^16^ | 2008 | \ | M | \ | \ | \ | \ | \ | \ | \ | \ | \ |
| 38 | Hirshberg ^16^ | 2008 | \ | M | \ | \ | \ | \ | \ | \ | \ | \ | \ |
| 39 | Hirshberg ^16^ | 2008 | \ | M | \ | \ | \ | \ | \ | \ | \ | \ | \ |
| 40 | Hirshberg ^16^ | 2008 | \ | M | \ | \ | \ | \ | \ | \ | \ | \ | \ |
| 41 | Hirshberg ^16^ | 2008 | \ | M | \ | \ | \ | \ | \ | \ | \ | \ | \ |
| 42 | Hirshberg ^16^ | 2008 | \ | M | \ | \ | \ | \ | \ | \ | \ | \ | \ |
| 43 | Hirshberg ^16^ | 2008 | \ | M | \ | \ | \ | \ | \ | \ | \ | \ | \ |
| 44 | Hirshberg ^16^ | 2008 | \ | M | \ | \ | \ | \ | \ | \ | \ | \ | \ |
| 45 | Hirshberg ^16^ | 2008 | \ | M | \ | \ | \ | \ | \ | \ | \ | \ | \ |
| 46 | Hirshberg ^16^ | 2008 | \ | M | \ | \ | \ | \ | \ | \ | \ | \ | \ |
| 47 | Hirshberg ^16^ | 2008 | \ | F | \ | \ | \ | \ | \ | \ | \ | \ | \ |
| 48 | Hirshberg ^16^ | 2008 | \ | F | \ | \ | \ | \ | \ | \ | \ | \ | \ |
| 49 | Hirshberg ^16^ | 2008 | \ | F | \ | \ | \ | \ | \ | \ | \ | \ | \ |
| 50 | Hirshberg ^16^ | 2008 | \ | F | \ | \ | \ | \ | \ | \ | \ | \ | \ |
| 51 | Hirshberg ^16^ | 2008 | \ | F | \ | \ | \ | \ | \ | \ | \ | \ | \ |
| 52 | Hirshberg ^16^ | 2008 | \ | F | \ | \ | \ | \ | \ | \ | \ | \ | \ |
| 53 | Hirshberg ^16^ | 2008 | \ | F | \ | \ | \ | \ | \ | \ | \ | \ | \ |
| 54 | Hirshberg ^16^ | 2008 | \ | F | \ | \ | \ | \ | \ | \ | \ | \ | \ |
| 55 | Hirshberg ^16^ | 2008 | \ | F | \ | \ | \ | \ | \ | \ | \ | \ | \ |
| 56 | Hirshberg ^16^ | 2008 | \ | F | \ | \ | \ | \ | \ | \ | \ | \ | \ |
| 57 | Hirshberg ^16^ | 2008 | \ | F | \ | \ | \ | \ | \ | \ | \ | \ | \ |
| 58 | Hirshberg ^16^ | 2008 | \ | F | \ | \ | \ | \ | \ | \ | \ | \ | \ |
| 59 | Hirshberg ^16^ | 2008 | \ | F | \ | \ | \ | \ | \ | \ | \ | \ | \ |
| 60 | Hirshberg ^16^ | 2008 | \ | F | \ | \ | \ | \ | \ | \ | \ | \ | \ |
| 61 | Hirshberg ^16^ | 2008 | \ | F | \ | \ | \ | \ | \ | \ | \ | \ | \ |
| 62 | Hirshberg ^16^ | 2008 | \ | F | \ | \ | \ | \ | \ | \ | \ | \ | \ |
| 63 | Hirshberg ^16^ | 2008 | \ | F | \ | \ | \ | \ | \ | \ | \ | \ | \ |
| 64 | Hirshberg ^16^ | 2008 | \ | F | \ | \ | \ | \ | \ | \ | \ | \ | \ |
| 65 | Hirshberg ^16^ | 2008 | \ | F | \ | \ | \ | \ | \ | \ | \ | \ | \ |
| 66 | Hirshberg ^16^ | 2008 | \ | F | \ | \ | \ | \ | \ | \ | \ | \ | \ |
| 67 | Hirshberg ^16^ | 2008 | \ | F | \ | \ | \ | \ | \ | \ | \ | \ | \ |
| 68 | Hirshberg ^16^ | 2008 | \ | \ | \ | \ | \ | \ | \ | \ | \ | \ | \ |
| 69 | Hirshberg ^16^ | 2008 | \ | \ | \ | \ | \ | \ | \ | \ | \ | \ | \ |
| 70 | Iida ^46^ | 2009 | Japan | M | \ | 55 | rectum | frontal lower gingiva | \ | chemotherapy | 11 | 11 | \ |
| 71 | Seoane ^47^ | 2009 | \ | M | \ | 59 | colon | mandible | \ | \ | \ | 24.3 | \ |
| 72 | Seoane ^47^ | 2009 | \ | F | \ | 70 | colon | hard palate | \ | \ | \ | 49 | \ |
| 73 | Soares ^3^ | 2010 | Brazil | M | \ | 42 |  | buccal gingiva between the left permanent mandibular canine tooth and the left permanent mandibular first premolar tooth | 35 | chemotherapy | \ | \ | first metastases |
| 74 | Favia ^48^ | 2010 | \ | F | \ | 66 | colon | edentulous retromolar area of the mandible | \ | \ | \ | \ | 180 |
| 75 | Favia ^48^ | 2010 | \ | F | \ | 35 |  | edentulous retromolar area of the mandible | \ | \ | \ | \ | 12 |
| 76 | Soda ^49^ | 2010 | \ | M | \ | 56 | rectum | retromolar pad | \ | surgery and chemoradiotherapy | 60 | \ | 24 |
| 77 | Landeyro ^50^ | 2010 | Spain | M | \ | 77 | sigmoid colon | teeth 1.5-2.1 (right upper jaw) | 30x40 | \ | \ | \ | 24 |
| 78 | Bhaskaran ^51^ | 2011 | UK | M | \ | 92 | sigmoid colon | left posterior dorsum of the tongue | 30 | palliative treatment | \ | \ | first metastases |
| 79 | Singh ^52^ | 2011 | India | F | \ | 42 | rectum | floor of the mouth | \ | chemotherapy and radiotherapy | \ | 0,8 | 39 |
| 80 | Amin ^53^ | 2011 | UK | M | \ | 75 | sigmoid colon | right mandibular region | 33 | palliative radiotherapy | \ | \ | 5 |
| 81 | Jham ^54^ | 2011 | USA | M | \ | 53 | colon | \ | \ | Surgery, chemotherapy | \ | \ | \ |
| 82 | Coad ^55^ | 2012 | \ | M | \ | 70 | rectum | right lower mandible | 50x60 | Trans-oral laser debulking of the right mandibular metastases, palliative care for symptomatic relief | 3 | 3 | first metastases |
| 83 | Murugaraj ^56^ | 2012 | UK | M | \ | 70 | rectum | lower right edentolous ridge and buccal mucosa | 30x30 | \ | \ | 9 | first metastases |
| 84 | He ^57^ | 2013 | China | M | \ | 67 | rectum | tonsil | \ | chemotherapy | \ | \ | 28 |
| 85 | Usman ^58^ | 2014 | \ | M | \ | 60 | rectum | left buccal mucosa, gingivo-buccal sulcus and lower alveolus | 77x101x6 | \ | \ | \ | first metastases |
| 86 | Yang ^59^ | 2014 | Taiwan | F | \ | 74 | rectosigmoid colon | area of the lower right first premolar | \ | palliative treatment | 3 | 3 | 24 |
| 87 | Yamauchi ^60^ | 2014 | Japan | M | \ | 50 | rectum | left lower gingiva | \ | chemotherapy | \ | \ | first metastases |
| 88 | Baranović ^61^ | 2015 | Croatia | M | \ | 78 | rectum | upper jaw | 30x20 | palliative care | 4 | 4 | 18 |
| 89 | Miyake ^62^ | 2015 | Japan | F | \ | 65 | transverse colon adenocarcinoma | second left lower premolar | 24x20 | surgery, chemotherapy and palliative radiation | \ | \ | 9 |
| 90 | Gill ^63^ | 2015 | USA | M | \ | 57 | rectum | right lateral tongue | 30 | surgery and radiotherapy | \ | \ | 24 |
| 91 | Watanabe ^64^ | 2016 | \ | M | \ | 64 | rectum | anterior lower mandibular gingiva | 26x21 | radiotherapy | \ | 4 | 84 |
| 92 | Kameta ^65^ | 2016 | Japan | M | \ | 73 | colon | buccal side of the right premolar region of the mandible | 12 | chemotherapy | \ | \ | 9 |
| 93 | Owosho ^2^ | 2016 | USA | F | \ | 77 | colon | Posterior mandible | \ | Radiotherapy | \ | 5 | \ |
| 94 | Owosho ^2^ | 2016 | USA | M | \ | 74 | colo-rectum | Mandibular ramus | \ | Radiotherapy | \ | 21 | \ |
| 95 | Owosho ^2^ | 2016 | USA | F | \ | 43 | colon | Anterior Maxilla | \ | Surgery + radiotherapy | \ | 21 | \ |
| 96 | Owosho ^2^ | 2016 | USA | M | \ | 59 | colon | Mandibular gingiva | \ | chemotherapy | \ | \ | \ |
| 97 | Ren ^18^ | 2017 | China | M | \ | 60 | colon | left gingival region of the mandible | \ | chemotherapy and radiotherapy | \ | \ | 25 |
| 98 | Romanet ^66^ | 2018 | \ | M | \ | 62 | colon | mandibular symphysis | \ | irradiation of 30 grays of the lesion | 15 | 15 | 84 |
| 99 | Di Stasio ^67^ | 2018 | \ | M | \ | 74 | colon | alveolar ridge and mucosa close to the first molar of the right upper maxilla | \ | \ | \ | 6 | \ |
| 100 | Salvador ^68^ | 2018 | Portugal | M | \ | 70 | lower rectum | trismus and left inferior wisdom toothache | 50 | palliative chemotherapy and radiotherapy | 2 | 2 | first metastases |
| 101 | Hadhri ^69^ | 2020 | \ | F | \ | 79 | right colon | \ | 60 | chemotherapy and radiotherapy | \ | \ | 60 |
| 102 | Neumann ^70^ | 2020 | \ | M | \ | 59 | colon | area of the incisors and tending to the hard palate | \ | surgery, chemotherapy and palliative radiotherapy | 10 | 10 | \ |
| 103 | Kolagatla ^71^ | 2022 | \ | F | \ | 74 | rectum | hard palate | 50 | radiotherapy and chemotherapy | 6 | 6 | \ |
| 104 | Ho ^72^ | 2022 | \ | M | \ | 60 | colon | gingiva and mandible | \ | \ | \ | \ | \ |
| 105 | Ho ^72^ | 2022 | \ | M | \ | 75 | colon | mandible | \ | \ | \ | \ | \ |
| 106 | Ho ^72^ | 2022 | \ | F | \ | 70 | colon | gingiva | \ | \ | \ | \ | \ |
| 107 | Ho ^72^ | 2022 | \ | F | \ | 92 | colon | gingiva | \ | \ | \ | \ | \ |
| 108 | Ho ^72^ | 2022 | \ | M | \ | 88 | colon | gingiva and mandible | \ | \ | \ | \ | \ |
| 109 | Ho ^72^ | 2022 | \ | M | \ | 57 | colon | gingiva and maxilla | \ | \ | \ | \ | \ |
| 110 | Ho ^72^ | 2022 | \ | F | \ | 81 | colon | mandible | \ | \ | \ | \ | \ |
| 111 | Ho ^72^ | 2022 | \ | F | \ | 85 | colon | gingiva and mandible | \ | \ | \ | \ | \ |
| 112 |  | 2023 | Italy | F | W | 38 | colon | mandible | 25x25 | chemotherapy and radiotherapy | 10 | 10 | 60 |

*Footnote*

Abbreviations: M, male; F, female; Nd, not defined; W, white; A, afroamerican; S, solitary; M, multiple; mos, months
